# Interface Reaction between Molten Al99.7 Aluminum Alloy and Various Tool Steels

**DOI:** 10.3390/ma14247708

**Published:** 2021-12-13

**Authors:** Maja Vončina, Tilen Balaško, Jožef Medved, Aleš Nagode

**Affiliations:** Department for Materials and Metallurgy, Faculty of Natural Sciences and Engineering, University of Ljubljana, Aškerčeva 12, 1000 Ljubljana, Slovenia; tilen.balasko@ntf.uni-lj.si (T.B.); jozef.medved@ntf.uni-lj.si (J.M.); ales.nagode@ntf.uni-lj.si (A.N.)

**Keywords:** tool steel–molten aluminum interaction, reaction layers, intermetallic phases, thermal analysis, thermodynamics

## Abstract

During the die-casting process as well as the hot forming process, the tool is subjected to complex thermal, mechanical, and chemical stresses that can cause various types of damage to different parts of the tool. This study was carried out to determine the resistance of various tool steels, i.e., UTOPMO1, HTCS-130, and W600, in molten Al99.7 aluminum alloy at a temperature of 700 °C. The formation kinetics of the interaction layer between the molten aluminum and tool steels was studied using differential scanning calorimetry. Light and field-emission scanning electron microscopy were used to analyze the thickness and nature of the interaction layers, while thermodynamic calculations using the Thermo-Calc software were used to explain the results. The stability of the HTCS-130 and W600 tool steels is better than the stability of the UTOPMO1 tool steel in the molten Al99.7 aluminum. Two interaction layers were formed, which in all cases indicate an intermetallic Al_13_Fe_4_ layer near the aluminum alloy and an intermetallic Al_5_Fe_2_ layer near the tool steels, containing small round carbides. It was confirmed that Ni reduces the activity of aluminum in the ferrite matrix and causes a reduction in the thickness of the intermetallic layer.

## 1. Introduction

During the die-casting process as well as the hot forming process, the tool is exposed to complex thermal, mechanical, and chemical stresses that can cause various types of damage to different parts of the tool. The most severe types of damage include wear, plastic deformation, thermal or mechanical cracks, and tool breakage. This results in higher financial costs and lower productivity and profits. Therefore, it is necessary to understand the interactions between the tool steel and molten aluminum alloy during the die-casting and/or hot forming process.

Anders Persson [1] claims that, in the case of the Al die-casting tool, product quality is affected not only by the resulting thermal cracks and wear, but also by the adhesion of the aluminum to the tool and erosion. Die casting is a cost-effective process for designing thin and complex walls with low tolerances and good surface quality. The advantages of such a casting process are high productivity, long tool life, low surface roughness, high material efficiency, and good mechanical properties.

Soldering is used in the high-pressure die-casting (HPDC) industry and results from the reaction between the die and the casting alloy. Chen et al. [2] demonstrated that, because of soldering, the solidified alloy can stick to the mold and form defective castings when ejected. Choi et al. [3] show that the remains of hard intermetallic phases can be deposited on the die surfaces and production must be stopped to remove the solder layer by polishing, which is costly in terms of the lost production time, the labor costs for polishing, and the reduction in the life of the die [4]. Therefore, soldering and its avoidance have been the subjects of considerable research efforts [5].

In this study, the high-temperature stability of the UTOPMO1 (AISI H11), HTCS-130, and W600 tool steels in molten Al99.7 aluminum was investigated. The chemical composition of the investigated steels is shown in Table 1. The UTOPMO1 tool steel is most frequently used in the group of hot work tool steels where the main alloying element is chromium, i.e., in chromium hot work tool steels. The UTOPMO1 tool steel has good hardenability from a relatively low austenitization temperature (about 1020 °C), good oxidation resistance, tempering resistance, erosion resistance with liquid aluminum, etc. [6]. After tempering, the optimum combination of hardness and ductility is achieved. This steel is used for hot forging tools, die casting tools, punching tools, and for the manufacture of knives.

For HTCS-130 and W600, Roberts et al. [7] have stated that it is difficult to determine to which group of hot work steels they belong according to the AISI standard, as both have 1.9 wt % W and 3.2 wt % Mo. In the ASM Handbook [8], it is written that this means that they do not belong to the group of tungsten hot work steels because of W (at least 8.5 wt % is required to belong to the group of tungsten hot work steels), and the same applies to Mo (at least 4.5 wt % is required to belong to the group of Mo hot work steels) [7]. The High Thermal Conductivity Steel (HTCS)-130 tool steel is a new generation of tool steel for hot work. The main purpose of its production was to increase the thermal conductivity. The HTCS-130 (Rovalma) tool steel is a hot work tool steel with very high thermal conductivity (up to 60 W/mK) that was developed especially for aluminum die casting. It is also used for other hot work applications such as other light metal die castings and solid forming or hot forming of coated sheets. In addition, it has proven to be advantageous for such applications as plastic injection molding or other applications that benefit from very high thermal conductivity and require high toughness [9]. Like most tool steels and other special steels with high thermal conductivity, Kaschnitz et al. [10] reported that HTCS-130 obtains its optimized mechanical and physical properties by appropriate heat treatment of the material before finishing. The W600 (Boehler) tool steel is mainly used for hot forming. It has good wear resistance, high thermal conductivity, and good weldability [11].

In order to develop a certain combination of mechanical properties, i.e., strength, hardness, and toughness, all tool steels must be properly heat treated before use. Heat treatment results in sufficient hardness, strength, wear resistance, and resistance to deformation at higher temperatures. Heat treatment consists of heating the material to the austenitization temperature, followed by austenitization where a certain holding time (the austenitization time) is needed to obtain a fully austenitic microstructure [12]. At this temperature, the dissolution of carbides and other intermetallic compounds also takes place; however, the dissolution rate depends on the austenitization temperature. Most of the carbides need to be dissolved in austenite in order to obtain fine carbides during tempering. Austenitization is followed by quenching (fast cooling) in order to initiate the austenite–martensite phase transformation. However, due to its low toughness and ductility, martensite needs to be tempered. With the tempering conditions (temperature and time), the properties of the tool steel before application are determined. Finally, after heat treatment the microstructure of the tool steel consists of tempered martensite with finely distributed carbides [13].

Mesquita [6] also demonstrated that, due to the solubility of iron in aluminum, the chemical reaction occurs, whereas intermetallic phases in certain stoichiometric proportions are formed according to the binary phase diagram for Fe–Al [14]. The most common phases arise from the binary phase diagram for Al–Fe, namely Al_5_Fe_2_ and Al_13_Fe_4_, which form a reaction layer [15]. For the formation of phases, two main conditions must be fulfilled, i.e., the optimal wetting and diffusion resulting from the difference in the chemical potentials of the elements in the solid tool steel and the melted aluminum. Tang et al. [16] reported that, at the phase boundary between the intermetallic phases and the solid metal, the atoms of aluminum and iron, using solid metal atoms, form new intermetallic phases. In this case, the phase boundary moves toward the solid metal. To achieve the appropriate mechanical and physical properties of the tools, the formation of these phases must be limited or prevented.

The system Al–Fe is characterized by a solid solution based on iron and six intermetallic compounds, namely Al_2_Fe, Al_5_Fe_2_, Al_3_Fe, AlFe, and Al_3_Fe_2_. There have been several studies on the microstructure of the interface layer between the molten aluminum and the tool steel, whereas AlFe_3_ and AlFe phases have a higher iron content, and therefore have better wear resistance [17]. In contrast, the Al_2_Fe, Al_5_Fe_2_, and Al_3_Fe phases contain a larger proportion of aluminum and are formed at temperatures below 1000 °C. These phases are fragile and therefore less persistent [18]. It was also shown that the phases at the interaction layer were Al_5_Fe_2_ and Al_3_Fe. Shahverdi et al. [19] proved that the latter grows faster and is the main phase, while in the later reaction stages it is easily detectable and partially dissolves in the molten aluminum. The reaction kinetics at the interface can lead to the formation of Al_5_Fe_2_ as the main component instead of Al_3_Fe, which is contrary to thermodynamic principles.

The thickness of the intermetallic layer rapidly grows when UTOPMO2 (AISI H13) tool steel and molten AlSi9Cu3 alloy are in contact [20]. Yan et al. [21] reported three intermetallic layers (Al_2_Fe, Al_5_Fe_2_, and Al_3_Fe) rich in aluminum at the interaction layer between the UTOP2 (AISI H21) tool steel and the AlSi9Cu3 (A380) alloy at 700 °C. It was proved that, regarding time, the thickness of the intermetallic layer increases, whereas the growth rates of the intermetallic phases vary [22]. The growth of the Al_5_Fe_2_ phase follows the parabolic law, which at a short testing time does not apply [23]. Initially, the growth of the phase boundary takes place linearly with respect to time based on the Al_3_Fe [24]. Shahverdi et al. [19] have shown that the rate of growth for the interface layer in a liquid aluminum–solid iron system follows a near parabolic distribution, but temperature plays an important role.

Furthermore, the other alloying elements, especially the alloying elements in the tool steel, such as chromium, molybdenum, silicon, manganese, and vanadium, are also present in the reaction layer. The thickness of the intermetallic layer is reduced in the presence of these elements; however, silicon has the greatest effect [24].

This investigation was conducted in order to establish the resistance of various tool steels, i.e., UTOPMO1, HTCS-130, and W600, in molten Al99.7 aluminum alloy at a temperature of 700 °C, which is a somewhat high temperature for molten aluminum alloy in the casting process. The results indicate only the high-temperature resistance of tool steels in contact with the molten aluminum alloy and do not include other factors such as erosion. The formation kinetics of the interaction layer between the molten aluminum and tool steels was investigated using differential scanning calorimetry (DSC). The thicknesses and the types of interaction layers were analyzed using light and field-emission scanning electron microscopy. In addition, thermodynamic calculations with the Thermo-Calc software were used to explain the influence of alloying elements of the tool steels on the activity of aluminum in the ferrite matrix and thus on the kinetics of the interaction layer’s growth. The most resistant investigated tool steel was identified.

## 2. Materials and Methods

To investigate the interaction between molten Al99.7 aluminum alloy and UTOPMO1, HTCS-130, and W600 hot-work tool steels in a tempered state, the isothermal DSC measurements were conducted at a temperature of 700 °C for 12 h. The measurements were carried out using an STA Jupiter 449C instrument from NETZSCH (NETZSCH Holding, Selb, Germany). The DSC measurements were performed in an argon atmosphere, while the temperature program was as follows: heating at 20 K/min to 750 °C, immediate cooling to the experimental temperature, which was maintained for 12 h, followed by cooling to a room temperature at 20 K/min. Samples from tool steels were prepared with dimensions of 4 mm in diameter and 1 mm in height. Samples from aluminum alloy were 4 mm in diameter and 3 mm in height. The surfaces of all samples were polished to ensure good contact between the steel and the aluminum alloy. The samples were placed in a corundum crucible, with the steel sample on the bottom of the crucible and the aluminum alloy sample on the top of the crucible.

After the DSC measurements, the samples were prepared metallographically. In order to analyze the thickness of the interaction layer of all samples, an Olympus BX61 light microscope was used. Furthermore, the composition of the interaction layers and the chemical composition of Fe-bearing phases were analyzed to identify the type of the phases that formed in the interaction layer using a Thermo Fisher Scientific Quattro S FEG SEM (ThermoFisher Scientific, Waltham, MA, USA) microscope with an Oxford Ultim^®^ Max EDS SDD EDS analyzer (Ultim® Max, Oxford Instruments, Abingdon, UK).

To identify the microstructural components present in the microstructure of the investigated steels, thermodynamic calculations were carried out with the Thermo-Calc software. The TCFE10 database (Thermo-Calc Software, 2020a, Thermo-Calc Software AB, Stockholm, Sweden) and Thermo-Calc version 2020a were used. The other reason for using Thermo-Calc was to calculate the thermodynamic stability of carbides present in the microstructure of the steel after tempering at 700 °C. In addition, the Thermo-Calc software tool was also used to simulate the effect of the alloying elements in the steels on the activity of aluminum in the ferritic matrix.

## 3. Results and Discussion

**Figure 1:**
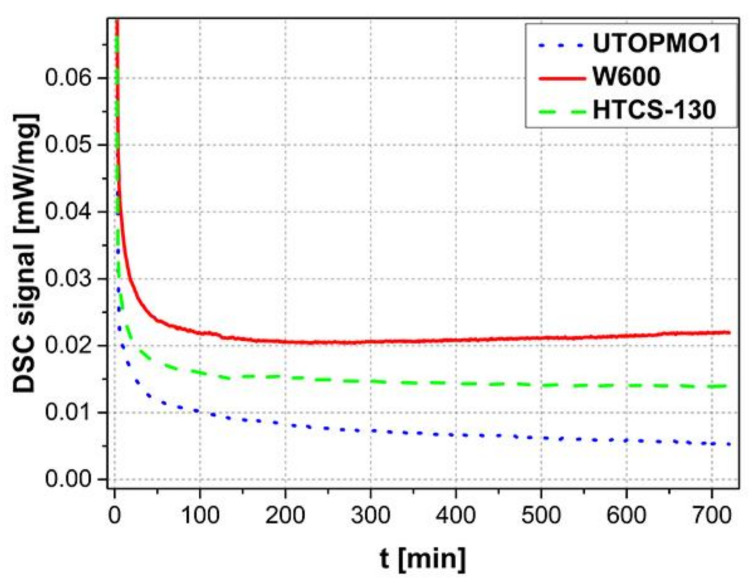
DSC curves of the dissolution of UTOPMO1, W600, and HTCS-130 tool steels in Al99.7 aluminum alloy at 700 °C.

Isothermal DSC curves of samples tested at 700 °C are shown in Figure 1. The dissolution of the UTOPMO1 tool steel is much more intensive when the HTCS-130 or W600 tool steel is dissolved in molten Al99.7 aluminum. In the beginning, the dissolution of all the experimental tool steels in the molten aluminum was very intensive; however, after about 250 s at 700 °C, the DSC curve stops falling and indicates the end of the dissolution or at least a slowing down of the dissolution. The slope of the curve of the W600 tool steel decreases and after 250 min it decreases slightly, while the slope of the curve of the other tool steels still decreases significantly.

**Figure 2:**
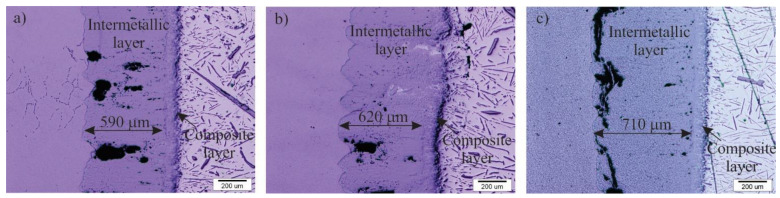
Micrographs of the interface between W600 (**a**), HTCS-130 (**b**), and UTOPMO1 (**c**) tool steel and Al99.7 aluminum alloy at 700 °C.

**Figure 3:**
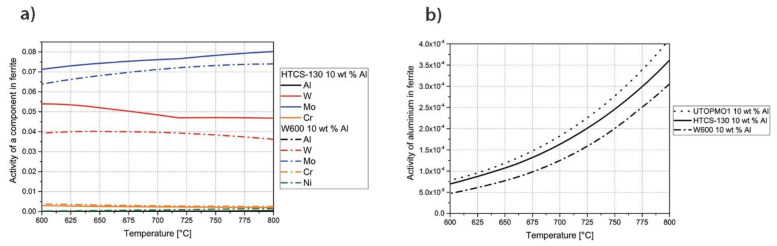
Effect of Ni on the activity of a component in ferrite for W600 and HTCS-130 (**a**) and the activity of aluminum in ferrite for W600, HTCS-130, and UTOPMO1 in which 10 wt % Al was considered (**b**).

The results from the DSC measurements were verified by optical and FEG SEM microscopy. The thickness of the interfacial layer with respect to the selected tool steel is marked in the light micrographs (Figure 2). The thickness of the intermetallic layer increases from the W600 tool steel via the HTCS-130 tool steel to the UTOPMO1 tool steel, which is consistent with the results of the DSC measurements. Shahverdi et al. [15] reported that the interface between the tool steel and Al99.7 aluminum alloy is composed of one intermetallic layer and a so-called composite layer. The intermetallic layer was thicker in the UTOPMO1 tool steel compared with the other two tool steels due to certain alloying elements in the steel. Persson [1] reported on the influence of alloying elements in the aluminum melt on the interaction layer’s thickness and this was also confirmed by other researchers [21]; however, this case shows the influence of alloying elements of the tool steel on the interaction layer’s thickness. The alloying elements of the tool steel, such as chromium, manganese, silicon, molybdenum, and vanadium, are present in the boundary layer (the reaction layer). These elements cause a reduction in the intermetallic layer’s thickness, with silicon having the greatest effect. Xiaoxia et al. [4] studied the role of titanium, while Shankar et al. [25] investigated the role of nickel in the thickness of the intermetallic layer and reported that titanium decreases and nickel increases the thickness of the intermetallic layer. The maximum thickness of the intermetallic layer was obtained in the UTOPMO1 tool steel (710 µm), while the thickness of the W600 and HTCS-130 tool steels was 590 and 620 µm, respectively. This can be explained by the effect of alloying elements on the activity of aluminum in the ferrite matrix (Table 2, Figure 3b), which was calculated by the Thermo-Calc software. The results show that the activity of aluminum in the ferrite matrix is highest in the UTOPMO1 tool steel, followed by the HTCS-130 and W600 tool steels. UTOPMO1 steel contains more silicon, chromium, manganese, and vanadium, while the main difference in chemical composition between W600 and HTCS-130 lies in the concentration of nickel (2.1 wt % vs. 0.04 wt %). The influence of nickel on the activity of aluminum in the ferrite matrix is clearly shown in Figure 3a. A higher aluminum activity reduces the equilibrium aluminum content in the ferrite matrix; therefore, the intermetallic layer is thicker. From this, it can be concluded that nickel reduces the activity of aluminum in the ferrite matrix; the result is a thinner intermetallic layer. The thickness of the composite layer is approximately the same in all three cases and is about 100 µm. Pores and cracks (black areas) are also visible in the oxide layers.

**Figure 4:**
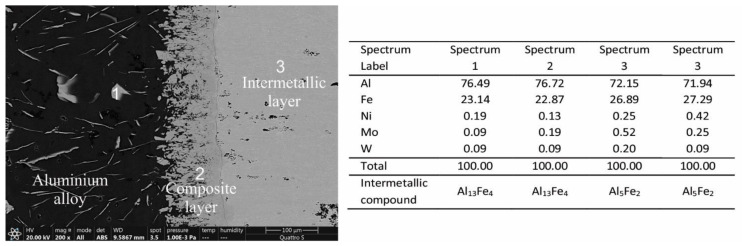
SEM micrographs of the interaction layer of the W600 sample tested at 700 °C in Al99.7 aluminum alloy. The corresponding EDS results are presented in at %.

**Figure 5:**
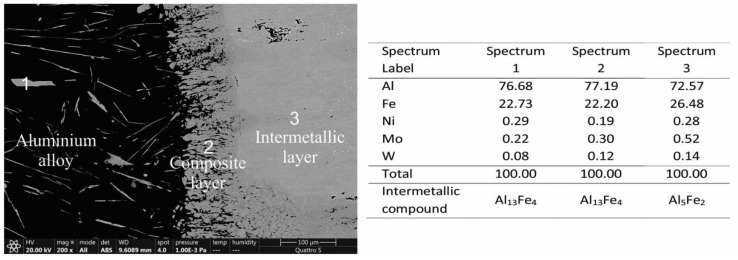
SEM micrographs of the interaction layer of the HTCS-130 sample tested at 700 °C in Al99.7 aluminum alloy. The corresponding EDS results are presented in at %.

**Figure 6:**
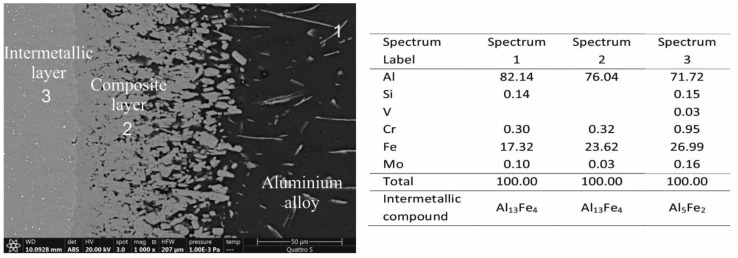
SEM micrographs of the interaction layer of the UTOPMO1 sample tested at 700 °C in Al99.7 aluminum alloy. The corresponding EDS results are presented in at %.

**Figure 7:**
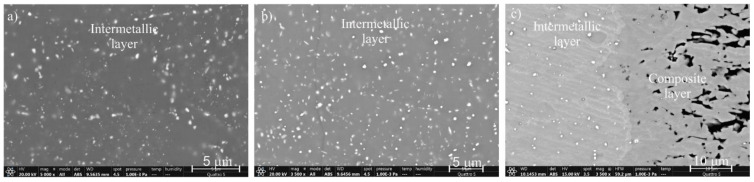
SEM micrographs of the interaction layer of the W600 (**a**), HTCS-130 (**b**), and UTOPMO1 (**c**) tool steel samples.

In addition, EDS analyses were performed on the interaction layers. As large an area as possible was captured and, in some areas, multiple results are presented. The results are as follows. In Figure 4, two interaction layers can be identified, indicating an intermetallic Al_13_Fe_4_ layer near the aluminum alloy and an intermetallic Al_5_Fe_2_ layer near the W600 tool steel. According to the FEG SEM/EDS analysis, this layer also contains small round particles, based on Mo and W, which are probably carbides of the M_6_C type (Table 3). In the interaction layers between the two other tool steels (HTCS-130 and UTOPMO1) and the Al99.7 aluminum alloy (Figure 5 and Figure 6), intermetallic layers of the same type as in the case of the W600 tool steel were identified. In the first case, the small particles in the intermetallic layer (Figure 7b) appear to be carbides based on Mo and W (type M_6_C, Table 3). In the second case where UTOPMO1 was tested (Figure 7c), they appear to be carbides as well (Table 3); however, they are based mainly on Cr (M_23_C_6_) and V (MC) as was also reported by Xu et al. [26].

However, before proceeding with the discussion of the metallographic analysis, the results of the thermodynamic calculations are presented to clarify which types of carbides were contained in the steels investigated (Table 4) and which carbides were stable at a temperature of 700 °C. All present carbides (Table 4) except for MC and M_7_C_3_ in UTOPMO1 were stable up to room temperature.

**Figure 8:**
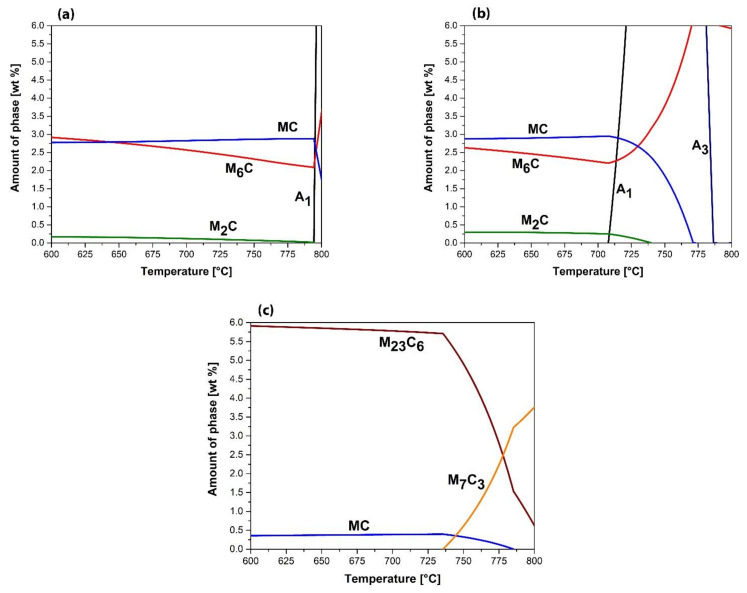
Thermodynamically calculated diagrams (amount of all phases vs. temperature) in the temperature range from 600 °C to 800 °C for HTCS-130 (**a**), W600 (**b**), and UTOPMO1 (**c**) tool steels.

Since the persistence of various tool steels in molten Al99.7 aluminum alloy was investigated at a test temperature of 700 °C, the thermodynamic calculations are only shown in the temperature range from 600 °C to 800 °C (Figure 8). In the case of the HTCS-130 steel, the A_1_ temperature is 794 °C (Table 4), and, as shown in the property diagram (Figure 8a), three types of carbides are stable at 700 °C: MC carbides, namely (Mo, W)C, M_6_C carbides, namely (Mo, Fe)_6_C, and M_2_C carbides, namely Mo_2_C. The amount of MC carbides and M_6_C carbides is approximately the same (2.82% and 2.56% by weight, respectively). On the other hand, the amount of M_2_C carbides is much lower (about 0.12 wt %). The property diagram of W600 steel (Figure 8b) shows a narrow range up to the A_1_ temperature, which is 708 °C (Table 4). At the temperature investigated, the three carbide types are thermodynamically stable as expected, as there are no significant differences in the chemical composition of the carbide formers in W600 steel compared with HTCS-130. Therefore, the M_6_C (Mo, Fe)_6_C, MC (Mo, W)C, and M_2_C (Mo_2_C) carbides are stable (Figure 8b). The amount of MC carbides and M_6_C carbides is still approximately the same (2.94 wt % and 2.24 wt %, respectively), but there is a larger difference compared with that in HTCS-130 steel. On the other hand, the amount of M_2_C carbides is much lower (about 0.26 wt %), but the amount is higher compared with HTCS-130. As far as the carbides are concerned, there should be no difference between W600 and HTCS-130 steel, but there is a risk that in the case of W600 steel we are on the edge of a two-phase range (ferrite + austenite). With the UTOPMO1 steel, the picture is completely different. The A_1_ temperature is 817 °C (Table 4). According to the property diagram of the UTOPMO1 steel (Figure 8c), two types of carbides are present that are thermodynamically stable at the temperature under investigation, namely M_23_C_6_ ((Cr, Fe)_23_C_6_) and MC (VC). The amount of M_23_C_6_ carbides is 5.78 wt % and the amount of MC carbides is 0.39 wt %; on the other hand, the M_7_C_3_ dissociation temperature is 736 °C. So, there is the possibility that the conversion of M_23_C_6_ to M_7_C_3_ has started, since M_7_C_3_ is more stable in the two-phase region (Figure 8c).

**Figure 9:**
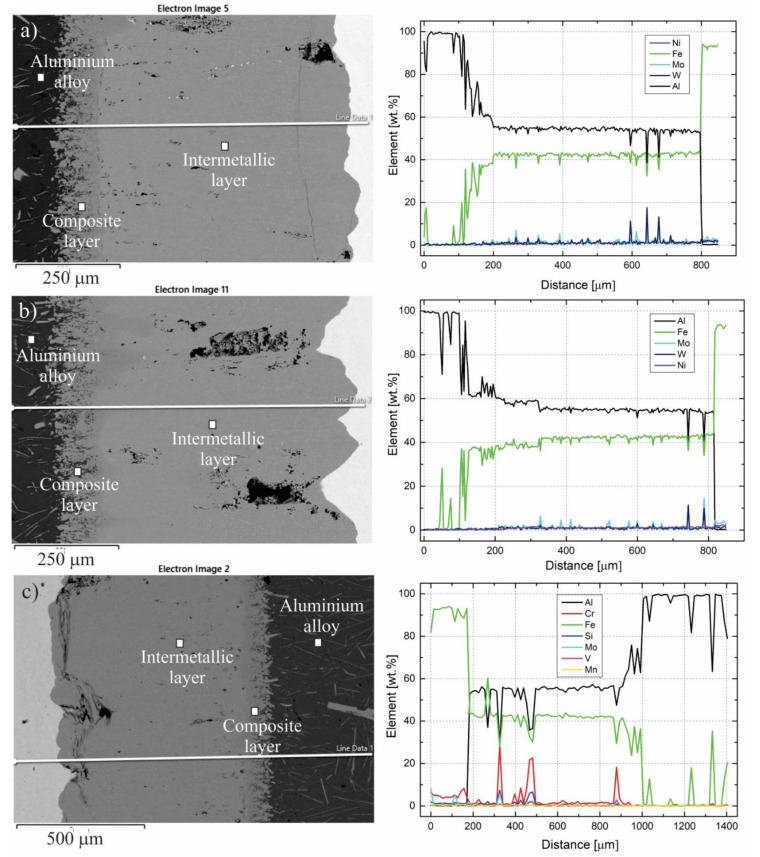
EDS line-scan analyses across the interface between W600 (**a**), HTCS-130 (**b**), and UTOPMO1 (**c**) tool steel and Al99.7 aluminum alloy.

In order to verify the different layers and the types of layers that formed between the different experimental tool steels and the Al99.7 aluminum alloy, line-scan EDS profiles across the interface were obtained and are shown in Figure 9. The analysis was performed on the straight line running across the interface. The distribution of the alloying elements presented in weight % is shown. According to the line-scan profile, the ratio between iron and aluminum in the layer close to the iron (the intermetallic layer) is the same in all cases, which indicates no influence of alloying elements in the tool steel on the type of interaction layer. The layer near the aluminum alloy also shows comparable results in the ratio between iron and aluminum in all three cases, with the concentration of iron increasing and the concentration of aluminum decreasing from the side of the aluminum alloy to the side of the intermetallic layer near the tool steel.

## 4. Conclusions

The stability of three different tool steels, namely W600, HTCS-130, and UTOPMO1, in molten Al99.7 aluminum alloy was investigated and the following conclusions were drawn.

The dissolution of the UTOPMO1 tool steel in molten Al99.7 aluminum alloy is much more intense compared with the HTCS-130 and W600 tool steel. The thickness of the intermetallic layer increases from the W600 tool steel to the HTCS-130 and UTOPMO1 tool steels. In all three cases, the interface between the tool steel and the Al99.7 aluminum alloy consists of an interaction layer and a composite layer. In the case of the UTOPMO1 tool steel, the intermetallic layer is thicker compared with the other two tool steels due to the higher activity of aluminum in the ferrite matrix. It was confirmed that nickel reduces the activity of aluminum and causes a reduction in the thickness of the intermetallic layer. The thickness of the composite layer was approximately the same in all three cases (100 µm).

The interaction layers were identified as an Al_13_Fe_4_, sometimes referred to as Al_3_Fe, intermetallic layer near the aluminum alloy and an Al_5_Fe_2_ intermetallic layer near the tool steels, which is also called composite layer. This layer also contains small round carbides. In the case of the W600 tool steel, the small particles in the intermetallic layer appear to be carbides based on Mo and W (M_6_C type); in the second case where UTOPMO1 was tested, they appear to be carbides based mainly on Cr (M_23_C_6_ type) and V (MC type).

## Figures and Tables

**Table 1:** Chemical composition (wt %) of the experimental tool steels.

Element	C	Si	Cr	Ni	V	W	Mn	Mo	Fe
W600	0.32	0.12	0.11	2.1	0.01	1.9	0.23	3.2	Rest
HTCS-130	0.31	0.07	0.1	0.04	0.01	1.9	0.08	3.2	Rest
UTOPMO1	0.36	0.97	5.05	0.09	0.38	0.01	0.54	1.22	Rest

**Table 2:** Activity of alloying elements in the investigated tool steels with different contents of aluminum (0, 1, and 10 wt %).

Steel Type	Activity of a Component in Ferrite				
	Al	Cr	Mo	W	Ni
W600/0 wt % Al	/	0.00496	0.05548	0.01984	0.01848
W600/1 wt % Al	2.63261·10^−7^	0.00499	0.05656	0.02091	0.01543
W600/10 wt % Al	1.24822·10^−4^	0.00288	0.07121	0.03973	8.11652·10^−4^
HTCS-130/0 wt % Al	/	0.00476	0.05417	0.01944	/
HTCS-130/1 wt % Al	2.99837·10^−7^	0.00474	0.05546	0.02054	/
HTCS-130/10 wt % Al	1.63116·10^−4^	0.00234	0.07614	0.04844	/
UTOPMO1/0 wt % Al	/	0.09993	0.03189	/	/
UTOPMO1/1 wt % Al	5.2121·10^−7^	0.09904	0.03391	/	/
UTOPMO1/10 wt % Al	1.8257·10^−4^	0.07716	0.00316	/	/

**Table 3:** EDS results of the carbides shown in Figure 7, presented in at %.

Sample/ Element	Carbides in Figure 7a	Carbides in Figure 7b	Carbides in Figure 7c
Al	1.58	63.86	54.45
Fe	49.18	23.77	12.23
Ni	0.65	0.48	
Mo	37.61	9.03	1.12
Si			0.19
Cr			27.49
V			4.51
W	10.98	2.87	
Total	100.0	100.00	100.00
Carbides based on:	Mo–W–Ni	Mo–W–Ni	Mo–V–C

**Table 4:** A1 temperatures and types of carbides and their precipitation temperature in the investigated steels.

Type of Steel	A1 Temperature/°C	Type of Carbide and Its Precipitation Temperature/°C				
		MC	M_2_C	M_6_C	M_7_C_3_	M_23_C_6_
HTCS-130	794	808	795	1095	/	366
W600	708	771	740	1101	/	364
UTOPMO1	817	1021	/	/	948	809

## Data Availability

Not applicable.
